# *GOModeler-* A tool for hypothesis-testing of functional genomics datasets

**DOI:** 10.1186/1471-2105-11-S6-S29

**Published:** 2010-10-07

**Authors:** Prashanti Manda, McKinley G Freeman, Susan M Bridges, TJ Jankun-Kelly, Bindu Nanduri, Fiona M McCarthy, Shane C Burgess

**Affiliations:** 1 Department of Computer Science and Engineering, Mississippi State University, MS, USA; 2 Institute of Digital Biology, Mississippi State University, MS, USA; 3 Life Sciences and Biotechnology Institute, Mississippi State University, MS, USA; 4 College of Veterinary Medicine, Mississippi State University, MS, USA

## Abstract

**Background:**

Functional genomics technologies that measure genome expression at a global scale are accelerating biological knowledge discovery. Generating these high throughput datasets is relatively easy compared to the downstream functional modelling necessary for elucidating the molecular mechanisms that govern the biology under investigation. A number of publicly available ‘discovery-based’ computational tools use the computationally amenable Gene Ontology (GO) for hypothesis generation. However, there are few tools that support hypothesis-based testing using the GO and none that support testing with user defined hypothesis terms.

Here, we present* GOModeler,* a tool that enables researchers to conduct hypothesis-based testing of high throughput datasets using the GO.* GOModeler* summarizes the overall effect of a user defined gene/protein differential expression dataset on specific GO hypothesis terms selected by the user to describe a biological experiment. The design of the tool allows the user to complement the functional information in the GO with his/her domain specific expertise for comprehensive hypothesis testing.

**Results:**

*GOModeler* tests the relevance of the hypothesis terms chosen by the user for the input gene dataset by providing the individual effects of the genes on the hypothesis terms and the overall effect of the entire dataset on each of the hypothesis terms. It matches the GO identifiers (ids) of the genes with the GO ids of the hypothesis terms and parses the names of those ids that match to assign effects. We demonstrate the capabilities of *GOModeler* with a dataset of nine differentially expressed cytokine genes and compare the results to those obtained through manual analysis of the dataset by an immunologist. The direction of overall effects on all hypothesis terms except one was consistent with the results obtained by manual analysis. The tool’s editing capability enables the user to augment the information extracted.* GOModeler* is available as a part of the AgBase tool suite (http://www.agbase.msstate.edu).

**Conclusions:**

*GOModeler* allows hypothesis driven analysis of high throughput datasets using the GO. Using this tool, researchers can quickly evaluate the overall effect of quantitative expression changes of gene set on specific biological processes of interest. The results are provided in both tabular and graphical formats.

## Background

As a result of the “genomic revolution”, high-throughput (HT) functional genomics experiments that measure the thousands of gene products (transcripts or proteins) in cells have become ubiquitous [1]. DNA sequencing technologies are commoditizing and democratizing transcriptome analysis by sequencing [2], further increasing the volume of transcriptome data. While getting the data is becoming less onerous, turning these massive datasets into knowledge, however, requires computational systems biology modelling approaches and is still challenging. One approach for modelling functional genomics datasets is to use canonical networks or pathways such as those provided by Ingenuity [3] and Ariadne [4]. A complementary approach is to take advantage of the Gene Ontology [5]. The GO has become the* de facto* standard for describing the molecular functions, biological processes, and cellular locations of gene products and is based on a structured, controlled vocabulary [5] that is computationally compliant.

In such a context, the GO may be used to identify which classes of gene products are represented, or over-represented, in functional genomics datasets. This has been done in one of three main ways: by “GO-Slimming”, by enrichment analysis or, less commonly, by hypothesis-testing. The GO Consortium provides pre-defined “GO Slim sets”, which are reduced representations of the three GO ontologies (biological process; molecular function; cell component) [5]. Use of GO Slims forces the analysis to be conducted at a pre-determined and very shallow conceptual level and thus does not make use of the full power of the GO.

At the time of writing there were at least 68 computational tools for GO enrichment analysis (GOEA) and these can be classified as belonging to one of three classes based on the algorithms used by each tool: singular enrichment analysis, gene set enrichment analysis, and modular enrichment analysis [6]. Regardless of their enrichment analysis classification, all GO enrichment analysis tools primarily focus on giving the user high level functional categories of GO terms significantly represented in the input gene set. Although useful to identify functional profiles of differentially-expressed gene products, neither GO Slims nor GO enrichment analyses methods support hypothesis-testing, especially based on user-specified hypothesis terms. While discovery-based approaches open up new areas and stimulate new hypotheses, HT functional genomics data should not be limited to discovery-based science. HT functional genomics data can be used, equally well as reductionist data, to test hypotheses.

The GO is ideal for hypothesis testing as it is designed to capture the explicit experimental data in the published literature in a computationally-amenable way. Initial tool development to support hypothesis testing using the GO include the* Gene Class Expression* tool [7] and eGOn [8]. The broad approach for these tools is to statistically determine if GO terms are differentially expressed between different gene data sets.

The adoption of GO-based functional genomics analysis tools has been slow because many assume a level of GO annotation of gene products that does not exist for many species, datasets or, microarray platforms, and/or require computer programming skills. Furthermore when the GO has been used for analyzing microarray data to find under- or over-represented GO terms associated with a dataset [5,9-13], these analyses are not based on the microarray quantitative values but rather on counts of GO terms. The results do not represent the true proportions of genes negatively- or positively-affecting a particular process or function.

Here we describe,* GOModeler,* a computational tool that does not compare gene lists against each other but rather is based on the hypotheses chosen by the user at the beginning of an experiment.* GOModeler* uses the GO, in combination with quantitative HT functional genomics data, to quantify the effects of input gene identifiers on the hypothesis terms. The results are presented in tabular and graphical formats. A qualitative table shows the individual effect of each gene on each hypothesis term and a quantitative table shows the overall effect of the entire gene dataset on each of the hypothesis terms. The net effects from the quantitative table are then summarised in a graphical output. The advantages of this tool are the following: 1) it allows the user to specify hypothesis terms; 2) it makes use of the detailed gene annotation and the hierarchical structure of the GO to determine effects; 3) it allows the user to supplement information obtained from the GO with their own expertise; and 4) it generates informative tabular and graphical summaries of the net effects of genes.

## Methods

### Input requirements

**Figure 1:**
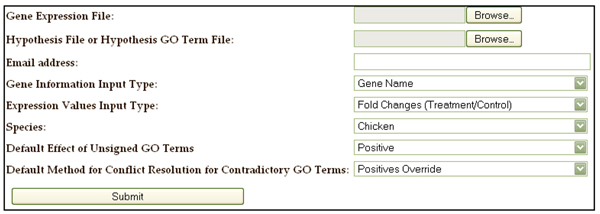
Interface of* GOModeler* showing the required inputs.

*GOModeler* requires the user to provide two input files as shown in Figure 1. The Gene Expression File is a text file containing a list of gene identifier/gene expression value pairs, one per line, with the identifier and the expression value separated by a comma. Identifiers accepted by* GOModeler* are UniProt accessions, gene names, and GenBank accessions. Alternatively, the Gene Expression File can contain sequences in FASTA format where the last line of each sequence is followed by a line containing a comma and an expression value. The gene expression values can be provided as fold changes (treatment/control), expression differences (treatment – control) or the logarithm of fold changes (log_2_ (treatment/control)). We assume that the gene expression values submitted by the user are a comparison of expression of a treatment and control or of two different conditions. If the data is from single-channel microarrays, we assume that the user has already processed the data to yield expression differences, ratios, or logarithms of ratios. For quantitative analysis, we require the following conditions to hold for the gene expression values:

1. Up-regulation is indicated by positive expression values.

2. Down-regulation is indicated by negative expression values.

3. No change in expression is indicated by a value of zero.

Expression values provided as fold changes are automatically converted to log_2_ (treatment/control) to ensure that these requirements hold.

**Figure 2:**
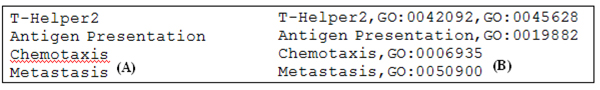
**File formats of input hypothesis files.** A. Example Hypothesis File; B. Example Hypothesis GO Term File

The second input file, the Hypothesis File, is a text file containing a list of hypothesis terms of interest to the user, one term per line (Figure 2a). The Hypothesis GO Term (HGT) Builder uses the Hypothesis File to create a Hypothesis GO Term (HGT) file as described below. If the user already knows which GO term identifiers are associated with each hypothesis term or if they have previously used the HGT Builder to create an HGT file, they can upload the HGT file directly. Each line of the HGT file contains a hypothesis term and a list of one or more associated GO terms separated by commas (Figure 2b).

**Figure 3:**
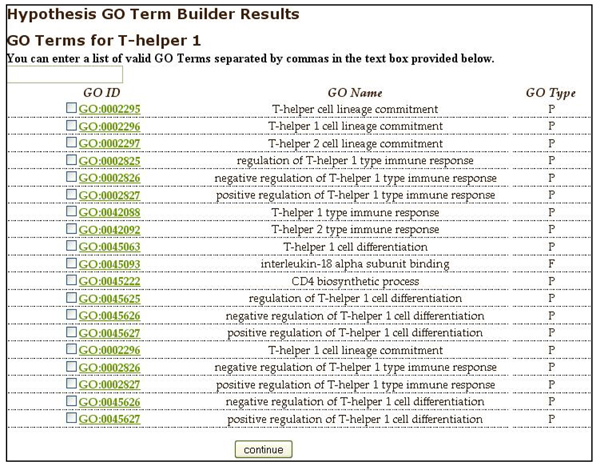
Hypothesis GO Term Builder interface. Users may select one or more terms and may enter one or more additional terms separated by commas in the text box. Clicking on a GO term takes the user to the Amigo page describing the GO term.

The Hypothesis GO Term Builder uses the Porter Stemmer [14][15] to extract stems for the hypothesis terms submitted by the user. For example, if the user submits *inflammation* as a hypothesis term, the stemmer would produce* inflam.* This enables matches to GO term names such as “*inflammatory response”*. The tool then searches all GO term names, descriptions, and synonyms for the specified hypothesis term stems. The results of the search are returned as a list of GO records with check boxes (Figure 3). The user can view additional details about each GO Term by clicking on the term name before making his/her choice. If there are no GO identifiers returned for a search term, the user is given a link to AmiGO [16], where they can conduct a more extensive search. The interface also provides a text box where the user can enter additional GO identifiers that were not found by the interface. The interface verifies that these additional GO identifiers are valid and not obsolete. The GO identifiers selected by the user or input in the text box are used to create the HGT file. After the file has been created, the user has the option of saving the file before starting the analysis.

Figure 1 also illustrates the additional information needed by* GOModeler.* The user is asked to enter their email address because the analysis conducted by* GOModeler* is time consuming for large datasets. The tool emails the results, a job identifier, and a link to the edit interface as soon as the analysis is complete. The results can be retrieved and edited through the edit interface.

**Figure 4:**
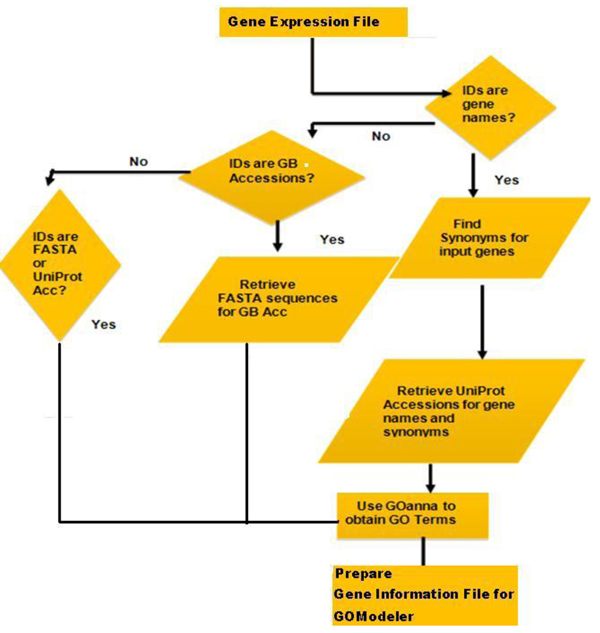
Process of obtaining GO identifiers for input identifiers.

*GOModeler* retrieves maximal GO annotation for each gene identifier listed in the Gene Expression File using the AgBase GOanna tool [13]. The process of creating the input file for GOanna varies depending on the type of gene identifier provided by the user as shown in the flow chart in Figure 4. GOanna accepts FASTA sequences or UniProt accessions. GenBank accessions are mapped to FASTA sequences and gene names are mapped to UniProt Accessions. GOanna conducts a BLAST search using standard GOanna parameters and retrieves GO identifiers for each input sequence. The user also needs to select the species of interest.* GOModeler* uses the species selected by the user to narrow the subsequent GOanna search to the most relevant species likely to have substantial GO annotation. For example, if the user selects “chicken”, GOanna searches chicken, mouse, and human databases. Table 1 gives a list of the species currently supported and the databases searched for each of these species. A Gene Information File is created containing the original information in the Gene Expression File along with the GO identifiers found for each gene. The user is also asked to specify a “Default Effect of Unsigned GO Terms” and “Default Method for Conflict Resolution of Contradictory GO Terms.” These parameters are discussed in detail in the following section.

**Table 1:** Species supported by GOModeler and the corresponding BLAST databases searched to obtain GO ids for gene identifiers.

Species	AgBase BLAST databases
Chicken	Chicken, Mouse, Human, Rat
Rat	Mouse, Human, Rat
Human	Mouse, Rat, Human
Mouse	Mouse, Human, Rat
Arabidopsis	Arabidopsis
Maize	Arabidopsis, Maize, Rice
Poplar	Arabidopsis, Poplar

### Mapping GO terms to effects

**Figure 5:**
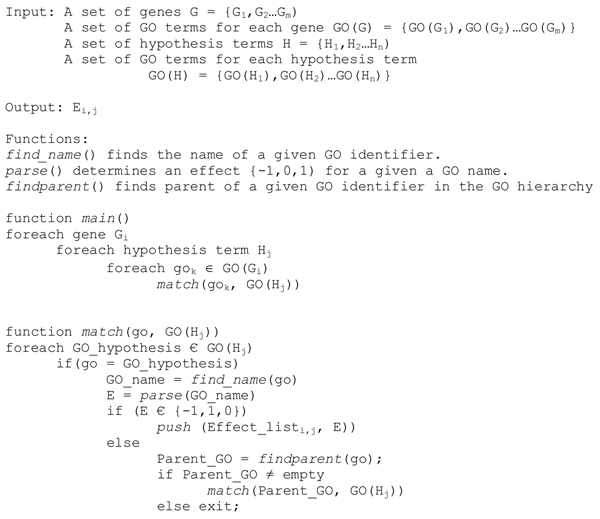
Algorithm to infer effects of GO terms from the GO terms for genes and GO terms corresponding to each hypothesis term.

The GO terms in the Gene Information File and the HGT file are the basis for the analysis conducted by* GOModeler.* The first step of the analysis is to match the GO identifiers for each gene in the Gene Information file to the GO identifiers related to each hypothesis term. Figure 5 describes the algorithm for the matching process. For each gene,* GOModeler* first matches each associated GO id with all the GO ids of each hypothesis term. If an exact match is found, the associated GO name is parsed to determine an effect value {+1, −1, 0} as described below. If no exact match is found, the match function recursively calls itself with the parents of the gene GO id and the hypothesis term GO id list as arguments to search for matches against generalizations of the gene GO term with the hypothesis GO terms. This operation is valid because the “true path rule” of the GO states that the path from a child to all its parents is always true- that is, if a particular GO term applies to a gene product, then all ancestors of that GO term also apply to the gene product [5].

After the matching process, GO term names are parsed to determine effects. Although the GO is a controlled vocabulary, the specific words “positive” and “negative” are not always used explicitly to describe effects. For example, the term GO:0002328 (*pro-B cell differentiation*) implies a positive effect while the terms GO:0006916 (*anti-apoptosis*), and GO:0044414 (*suppression of host defenses*) imply negative effects. If a name contains the words* positive, pro, stimulates, increases/d or upregulates*, an effect value of +1 is assigned. If the name contains the words* negative, anti, suppresses, inhibits, decreases/d* or* downregulates,* an effect of −1 is assigned. If the GO term does not specify either positive or negative effects, the assigned effect will depend on the “Default Effect of Unsigned GO Terms” specified by the user. If the user selects “no effect” then the final effect for “unsigned terms” will be 0. If the user selects “positive” all matched terms where* GOModeler* cannot assign a positive or negative effect are assumed to have a positive effect (+1). For example, the term GO:0042981 (*regulation of apoptosis*) is an unsigned term that could be assumed, based on common usage in manuscripts, to have a positive effect. This problem is created, and needs to be accounted for, because GO biocurators annotate only explicitly written facts and do not infer further [17].

In some cases, GO terms with contradictory effects will be found for the same gene-hypothesis term pair. This is quite reasonable as gene product effects are commonly context dependent. Regardless, in this case, the conflict resolution method specified by the user is used to determine the effect. Conflict resolution methods supported are: positives override, negatives override, and the greater of positives and negatives. At the conclusion of the matching process,* Effect_list_i,j_* will contain all of the effects found for gene* i* and hypothesis term* j.*

### Summarizing effects

**Figure 6:**
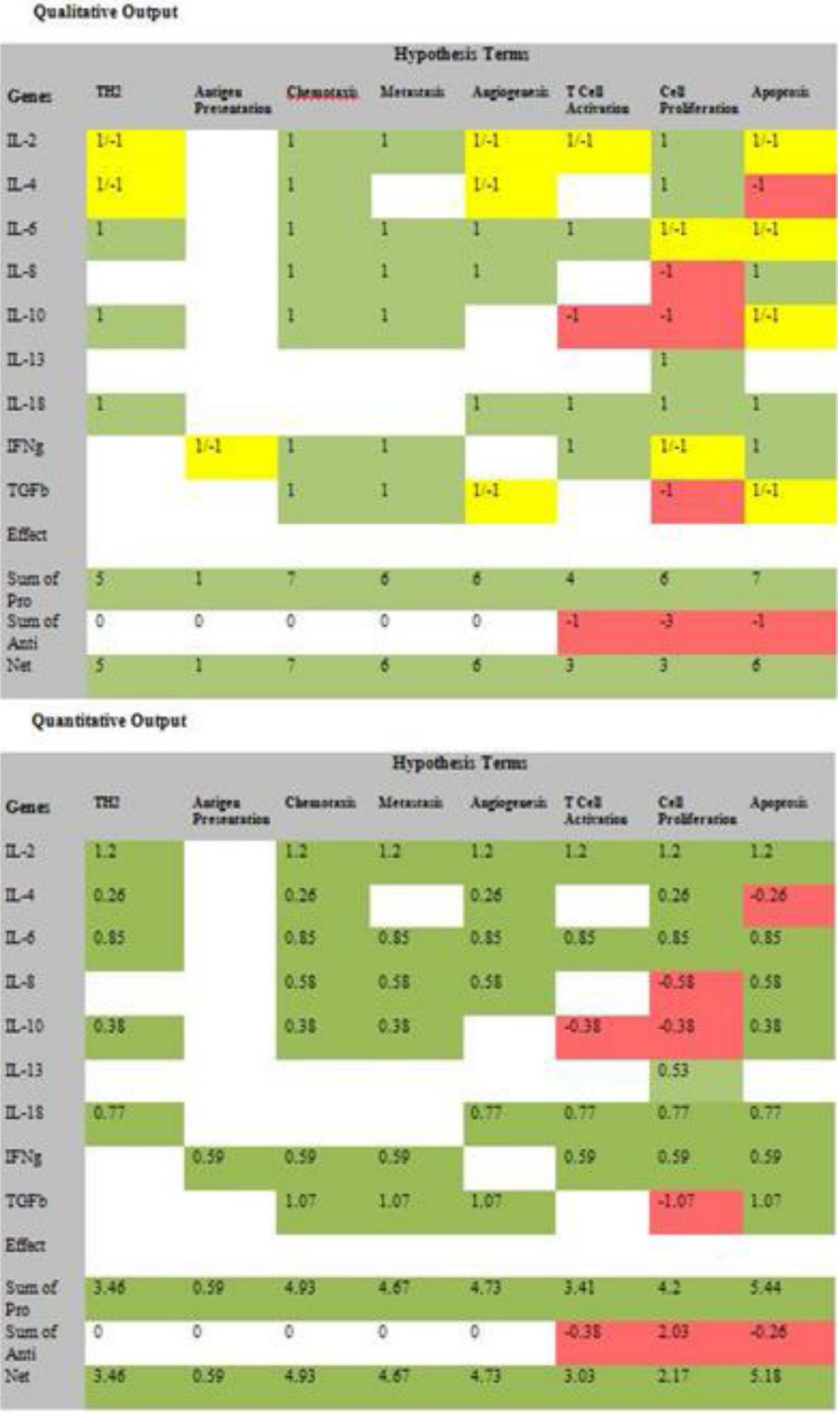
Qualitative and quantitative tabular results for* GOModeler* for the test cytokine dataset.

*GOModeler* generates a qualitative table, a quantitative table (Figure 6) and a graphical summary of the net effects of the gene set on each of the hypothesis terms. The qualitative table depicts the individual qualitative effect QL_i,j_ of each gene G_i_ on each of the hypothesis terms H_j_ where QL_i,j_ ∈ {−1, 0, +1, *undefined*}. Positive effects are highlighted in green and negative effects are highlighted in red. If QL_ij_ is defined, the quantitative effect of each gene is computed as:

*QT_i,j_* = QL_i,j_*GE_i_ where* GE_i_* is the gene expression value associated with gene* G_i_*.

The net quantitative effect,* N_j_,* on each hypothesis term, *H_j_*, is computed as:

The quantitative table also displays the total pro, anti and net effects of the gene set on each hypothesis term (Figure 6).

**Figure 7:**
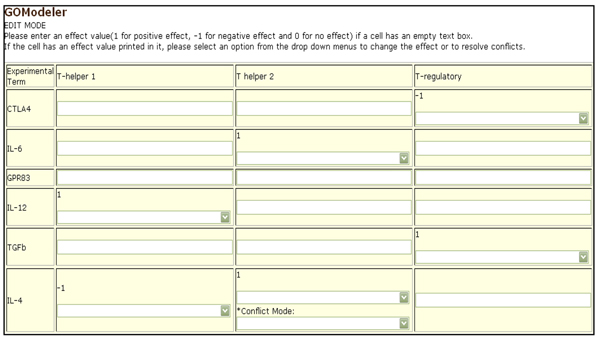
Edit Interface. This interface allows the user to modify term effects and to enter effects when the tool found none.

**Figure 8:**
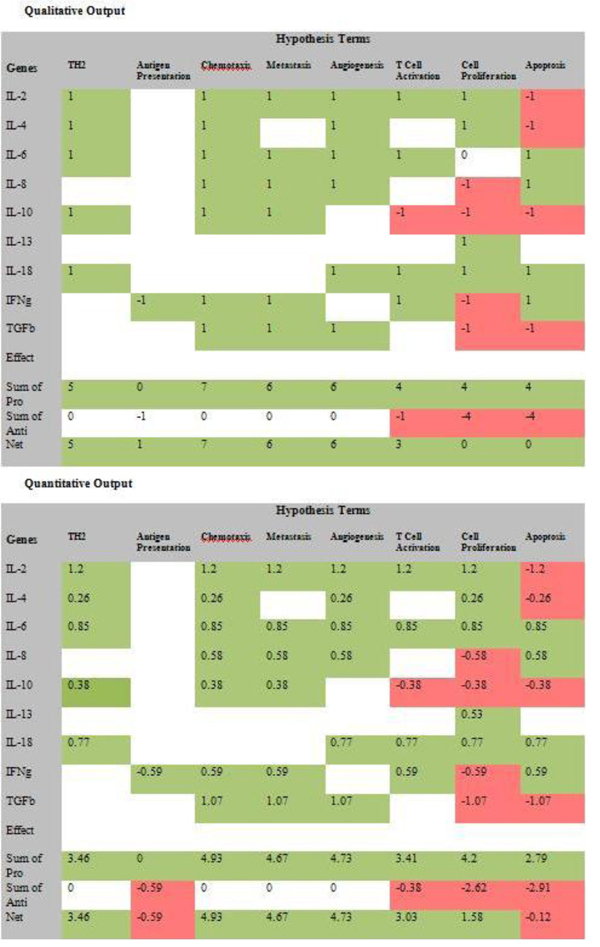
Qualitative and quantitative tabular results for* GOModeler* for the test cytokine dataset after conflict resolution by domain user.

**Figure 9:**
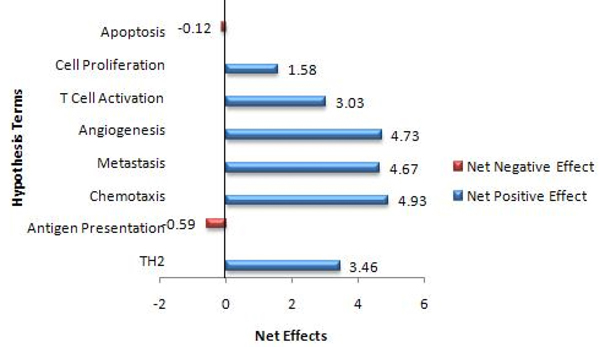
Graphical summary of net effects from the quantitative output in Figure 8.

The tool is designed to allow the user to incorporate his/her domain knowledge to augment the information encoded in the GO. The interface allows the users to change the effect values obtained by* GOModeler* or add an effect value where the tool was not able to determine an effect (Figure 7). The users can use the Job ID sent by* GOModeler* to view the results in the edit interface. The edit interface also allows users to resolve conflicting effects resulting from different GO terms for the same gene identifier. For example, Rat IL-6 (*UniProt:* P20607) is annotated to GO:0008285 (*negative regulation of cell proliferation*) with an evidence code of IMP (Inferred from Mutant Phenotype) but it is also annotated to GO:0008284* positive regulation of cell proliferation* with an evidence code of IS0 (Inferred from Sequence Orthology) [16]. In such a case, the tool infers both positive and negative effects and it is up to the user to investigate the source of the conflict and change the effects based on the experimental context. Figure 8 shows the results after the domain expert resolved conflicts based on knowledge of the experimental context of these results. The tool summarizes the net effect of the dataset on each hypothesis term in a graphical format (Figure 9) where the colour coding is consistent with the colour coding in the qualitative and quantitative tables.

## Results and discussion

Data from two cancer biology studies with the aim of defining the phenotype of specific populations of neoplastically-transformed cells were used to demonstrate the utility of *GOModeler *[18]. The* GOModeler* results from the first study involving a set of nine differentially-expressed cytokine genes are shown in Figure 6. Results for the second study involving ten differentially-expressed genes are given in Additional file 1. For the *GOModeler* analysis, the* species* selected was* chicken,* the* gene input type* was* gene name* and the other parameters selected were* positive* as the option for* default effect for unsigned GO terms* and* positives override* as the option for* conflict resolution.* Table 2 shows a comparison of the qualitative effects obtained using* GOModeler* and the results obtained by manual analysis by a PhD level immunologist. The direction of the net effects obtained by* GOModeler* is in agreement with 75% of the results obtained by the manual analysis by an immunologist. The results from* GOModeler* and the manual analysis differ for the hypothesis terms* apoptosis and antigen presentation* and inspection of the results in edit mode reveals that two of the entries (IL-6 and IL-10) had conflicting effects (−1 and +1) for* apoptosis.* Because we had selected* positives override* as the conflict resolution mechanism, the tool had selected +1 over −1. The user has the ability to change the effect for individual cells in edit mode.

**Table 2:** Comparison of GOModeler results for the test cytokine dataset with manual analysis results. Columns with the heading G were generated by GOModeler and those with the heading of M were obtained by manual annotation by an immunologist.

Genes	Hypothesis Terms															
	**TH2**		**Antigen Presentation**		**Chemotaxis**		**Metastasis**		**Angiogenesis**		**T Cell Activation**		**Cell Proliferation**		**Apoptosis**	
	
	**G**	**M**	**G**	**M**	**G**	**M**	**G**	**M**	**G**	**M**	**G**	**M**	**G**	**M**	**G**	**M**

**IL-2**	1/-1			0	1		1		1/-1		1/-1	1	1	1	1/-1	-1

**IL-4**	1/-1	1		1	1	1			1/-1				1	1	-1	0

**IL-6**	1			0	1	-1	1	0	1		1		1/-1	0	-1/1	

**IL-8**					1	1	1	1	1	1			-1	-1	1	

**IL-10**	1	1		1	1		1	0			-1		-1	-1	-1/1	-1

**IL-13**		1											1	0		

**IL-18**	1	1						1	1	1	1		1	1	1	1

**IFNg**		-1	-1	1	1		1				1		1/-1	-1	1	1

**TGFb**		1			1		1		1/-1				-1	1	1/-1	-1

**Effect**																

**Sum of Pro**	5	5	0	3	7	2	6	2	6	2	4	1	6	4	7	2

**SumofAnti**	0	-1	-1	0	0	-1	0	0	0	0	-1	0	-3	-3	-1	-3

**Net**	5	4	-1	3	7	1	6	2	6	2	3	1	3	1	6	-1

Other differences between the manual scoring and* GOModeler* can be attributed to incompleteness of the GO [18]; that is, published data exists and GO terms exist but the two have not been linked by GO biocurators. For example, it is obvious that our immunology domain expert has brought substantial knowledge to bear about the hypothesis term* antigen presentation* that is not yet annotated and present in the GO databases for these genes. In some cases, such as the effect of IL-6 on* chemotaxis, GOModeler* and the domain expert found opposite effects. Manual inspection of the GO annotation of IL-6 confirms that* GOModeler* is obtaining the correct effect based on information available in the GO annotation of IL-6 for rat, mouse, human and chicken. This is a specific example where the GO is incomplete and also where the effect is context dependent and so the gene product effect can be positive in some cases and negative in others.

In general,* GOModeler* tends to identify more positive effects than negative effects. This phenomenon can occur when genes have conflicting positive and negative (pro and anti) effects specified by the GO (indicated by “(+1/−1)” in the qualitative table) and, additionally, a bias is introduced by selecting “positives override” for conflict resolution. This is obviously something that users must be aware of and it is reasonable to assume that they will be. However, there are more complicated factors also. For example most GO terms are “unsigned”, i.e. they do not indicate positive or negative. We have opted to use “positive” as the default effect for unsigned GO terms based on colloquial usage and our experience that “positive effects” are often implicit. An example is the physical manifestation of programmed cell death known as* apoptosis.* “*positive regulation of apoptosis*” (GO:0043065) and “*negative regulation of apoptosis*” (GO:0043066) can be used to indicate pro and anti effects by annotators. However, authors can imply that a gene positively regulates apoptosis without explicitly saying so. In such cases the GO term “*apoptosis*” (GO:0006915) is used for annotation and yet the domain specific experts will know that this is a positive regulation. Finally, scientists tend to publish their positive data and to make hypotheses in a positive sense.

Unlike most GO-based discovery tools [6] that focus on the under- or over-representation of GO categories,* GOModeler* supports hypotheses testing using the GO. Although modern high throughput methods support discovery-based science, hypothesis driven science remains the approach used by most molecular biologists and required for funding from many agencies (e.g. NIH). GOEA tools can be used to generate an initial list of hypotheses, but they are of limited value for hypothesis testing. GOEA tools can typically only identify hypotheses for GO terms that are over and underrepresented in a dataset. These statistical approaches are not applicable for analysis of biological processes that involve only a few genes because the GO terms involved occur in such small numbers that they will never be identified as “over-or under-represented” by statistical analysis. By contrast* GOModeler* can identify such effects. In addition, most GOEA tools limit their analysis to a specific and arbitrary level of the GO DAG (i.e. GO Slim categories). These categories are often so general that they can be of little use in hypothesis-driven research. Some GOEA tools allow the user to “drill down” from the GO Slim categories, but as the GO terms become more specific, there will be fewer genes with these annotations, making it highly unlikely that they will pass the statistical tests for under- or over- representation. In addition, selection of an arbitrary level of detail falsely assumes that all terms at the same level in the GO DAG hierarchy are at the same conceptual level [18]. In addition, some parts of the GO are much better developed than others. Tools that focus on over- or under-represented GO terms provide no information about the direction of effect of the dataset on the categories. Although* GOModeler* is a GO-based tool, the issues addressed by* GOModeler* and GOEA tools are fundamentally different and each has their own uses.

To illustrate the differences in* GOModeler* and GOEA tools, we have used the dataset in Figure 6 with three popular GOEA tools, AgriGO, GOStat and DAVID. GOStat and DAVID reported only high level terms (e.g. immune response, extracellular region, abiotic response to stress) and did not “discover” any of the hypothesis terms in our dataset. With our small set of genes, AgriGO generates a message stating that “Sorry, less than 10 entries can be mapped with GO. Analysis Failed.”* GOModeler,* on the other hand, does not rely on statistical over or under representation and allows users to control the level of specificity appropriate for testing their hypotheses.

One limitation that* GOModeler* shares not only with GOEA tools but also with network and pathway tools such as Ingenuity Pathway Analysis (http://www.ingenuity.com/) is that the computed quantitative effects provide a simplified view of gene effects. All of these methods ignore complexities introduced by differential gene effects on gene pathways, biological processes and molecular functions (though they can take cell location into account). Adding to this complexity is the contextual relative effect of a gene product. This view does, however, allow us to show the direct effects of relative expression between two comparable systems (control versus treatment) i.e. genes that are much more highly expressed in the treatment system will have higher quantitative effects compared with the control system. We have already successfully applied this approach in several published papers that used a preliminary version of* GOModeler* with substantial user input [19-21].

*GOModeler* is not a gene expression analysis tool and an essential underlying assumption of* GOModeler* is that appropriate statistical analysis of differential gene product expression has been done. This is completely compatible with reductionist approaches and* GOModeler*’s utility is to quickly survey the GO to assign terms from one of the three ontologies based on the user’s hypothesis terms at the most appropriate and granular level of the GO. For example, there are currently only six genes annotated in the GO to be involved in* angiogenesis.* Reductionist biologists could test a hypothesis about genetic regulation of angiogenesis by, for example, quantitative PCR of these six genes. Although we often think of HT methods as associated with discovery based-science, a HT functional genomics experiment (such as RNAseq) would also measure the same six mRNAs and could be used for hypothesis driven research. As demonstrated by our examples,* GOModeler* can be used for the reductionist approach [20,21] or for a HT functional genomics approach [19,22-24]. HT functional genomics experiments, however, allow many other genes to be tested for other hypotheses using* GOModeler.*

## Conclusions

*GOModeler* facilitates hypothesis testing from HT functional genomics datasets. The results obtained from* GOModeler* demonstrate that the tool is a valuable resource for hypothesis-driven research and that it provides capabilities complementary to other tools that focus on enrichment analysis or statistical comparison of two datasets. Because there are problems with the incompleteness of the GO and context-dependent effects of gene products on biological processes, molecular functions, and cellular location, we provide the user with the ability to supplement the automated results with his/her expertise through editing.

### Availability and requirements

*GOModeler* is available for public access at the* AgBase* website http://agbase.msstate.edu/ → Tools →* GOModeler. GOModeler* has been implemented in Perl and HTML. Results are provided in both tabular and graphical format. Help documentation is available from to assist users in preparation of datasets, interpretation of result and use various tool features.

## Competing interests

No competing interests exist.

## Authors' contributions

MF wrote the original version of* GOModeler.* PM has refined the tool, compiled the datasets used for evaluation, participated in the optimization and performance evaluation of the code and drafted the manuscript. SCB conceived the pipeline; supplied datasets used in the manuscript, conducted the manual analysis of the dataset, and helped write the manuscript. FM helped develop the strategy for finding GO terms for gene products and hypothesis terms, in evaluating results, and in writing the paper. SMB participated in the pipeline and algorithm development and helped write the manuscript. TJK developed the format and code for generating the tabular and graphical output. BN evaluated the tool and provided suggestions for improvement. All authors have read and approved the manuscript.

## Supplementary Material

Additional File 1 Title of data: Figure 1 Qualitative and quantitative tabular results for* GOModeler* for the second cancer biology study. Figure 2 Graphical summary of net effects. Click here for file
