# Supplementary material for: GOModeler- A tool for hypothesis-testing of functional genomics datasets
Source: BMC Bioinformatics. 2010 Oct 7;11(Suppl 6):S29. doi: 10.1186/1471-2105-11-S6-S29 (PMC3026376; doi:10.1186/1471-2105-11-S6-S29)
Supplement: Additional File 1 — Title of data: Figure 1 Qualitative and quantitative tabular results for GOModeler for the second cancer biology study. Figure 2 Graphical summary of net effects. [file 1471-2105-11-S6-S29-S1.pdf]

Figure 1

Qualitative and quantitative tabular results for *GOModeler* for the second cancer biology study [1].

Qualitative Output

	Hypothesis Terms			
Genes	TH-1	TH-2	T-reg	Inflammation
CTLA4			1	
IL-6		1		1
IL-12	1			
TGFb			1	1
IL-2	1	-1	1	1/-1
IL-10	-1	1		1/-1
IL-13				1
IL-4	-1	1/-1		
IL-8				1
IL-18	1	1		
Effect				
Sum of Pro	3	4	3	6
Sum of Anti	-2	-1	0	0
Net	1	3	3	6

Quantitative Output

	Hypothesis Terms			
Genes	TH-1	TH-2	T-reg	Inflammation
CTLA4			-0.94	
IL-6		0.26		0.26
IL-12	-0.47			
TGFb			0.12	0.12
IL-2	3.05	-3.05	3.05	3.05
IL-10	2.39	-2.39		-2.39
IL-13				0.22
IL-4	-0.26	0.26		
IL-8				0.25
IL-18	0.91	0.91		
Effect				
Sum of Pro	6.35	1.43	3.17	3.9
Sum of Anti	-0.73	-5.44	-0.94	-2.39
Net	5.62	-2.58	2.23	1.51

Figure 2

Graphical summary of net effects from the quantitative output in Figure 1.

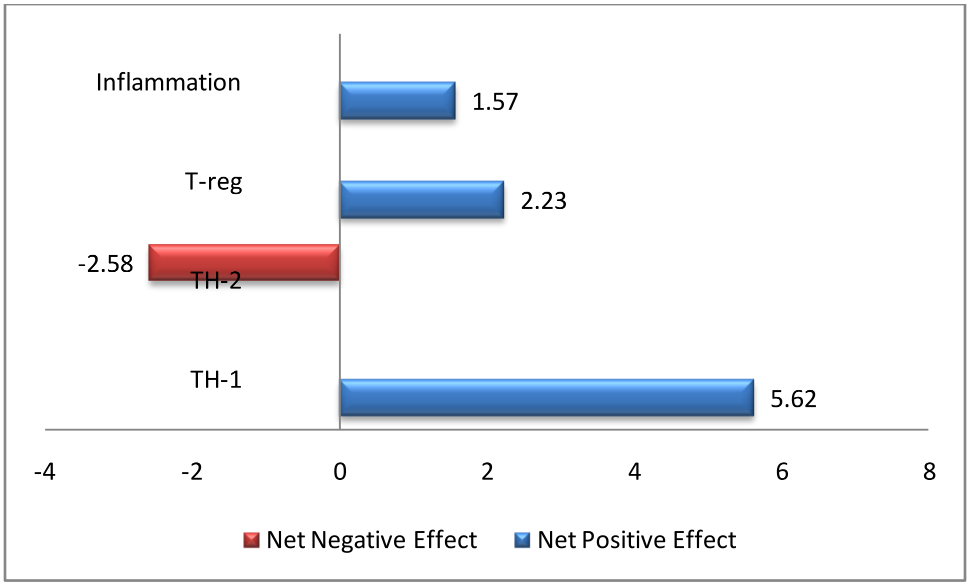

References

1. **Shack LA, Buza JJ, Burgess SC: The neoplastically transformed (CD30hi) Marek's disease lymphoma cell phenotype most closely resembles T-regulatory cells. *Cancer Immunol Immunother* 2008, 57(8):1253-1262.**
